# The Zoonosis Council: establishing clarity on zoonotic diseases for the general public

**DOI:** 10.1093/af/vfaf037

**Published:** 2025-10-14

**Authors:** Luke Sanders, Mamie-Cate Haydon, Riley Messman

**Affiliations:** Animal and Food Sciences Department, Oklahoma State University, Stillwater, OK; Animal and Food Sciences Department, Oklahoma State University, Stillwater, OK; Animal and Food Sciences Department, Oklahoma State University, Stillwater, OK

**Keywords:** communication, one health, social media, zoonotic diseases

**Implications**

- Engaging veterinarians, animal scientists, and public health professionals in proactive, collaborative communication can help combat misinformation and restore public confidence in their expertise. This is vital for informed decision-making during zoonotic disease outbreaks, preventing unnecessary panic and promoting science-based responses.
- Establishing a centralized, reliable source for zoonotic disease information can prevent agricultural and livestock industry losses due to public fear. It also ensures individuals follow appropriate health measures, rather than responding impulsively to sensationalized media narratives.
- Through social media, media partnerships, and transparency, the animal health sector can shift the conversation on zoonotic diseases from a reactive stance to a proactive one. This approach leads to a more knowledgeable and prepared public, reducing confusion and increasing confidence in public health measures.

## Introduction

Zoonotic diseases pose a major threat to both animal and human health, but misinformation spreads rapidly, often overshadowing expert voices. The 2023–2025 Highly Pathogenic Avian Influenza (HPAI) epidemic in the United States (USDA-APHIS, 2024) highlighted how misinformation can fuel panic, misguide public health responses, and unfairly target agricultural producers. As zoonotic outbreaks increasingly intertwine with media narratives and political agendas, the need for a coordinated, reliable, and science-driven communication strategy is critical for advancing One Health goals.

This paper proposes the creation of a Zoonosis Council, a collaborative body composed of veterinarians, scientists, influencers, Extension professionals, public health experts, and industry leaders. This Council will serve as a strategic hub for clear, timely communication during both outbreaks and nonemergency periods. Similar approaches in other nations, such as the United Kingdom’s Human Animal Infections and Risk Surveillance group, and in other fields, like the United States’ Centers for Disease Control and Prevention’s (CDC) Epidemic Intelligence Service, offer proven models for such a proactive, interdisciplinary initiative. While the CDC focuses on public health surveillance and response, the proposed Zoonosis Council will emphasize science communication and stakeholder engagement tailored primarily to U.S. audiences.

## Purpose and Audience

The proposed Zoonosis Council is designed to serve a broad range of audiences, including:

- Producers and agricultural stakeholders needing accurate, rapid outbreak guidance.
- Consumers, to mitigate fear and maintain trust in food systems.
- Decision-makers and policy leaders, for timely science-backed data.
- Media professionals and influencers, who can amplify correct information across platforms.

## Role of the Zoonosis Council

The Zoonosis Council will act as a leading private agriculture-focused consultant and information hub for zoonotic disease prevention, management, education, and crisis communication. During outbreaks, it will provide real-time updates, expert insights, and preventive measures to combat misinformation and reduce public confusion (Sell, 2017). In non-outbreak periods, it promotes awareness through ongoing education and One Health initiatives (WHO, 2024). The Council will also seek to align its efforts with recent federal initiatives, including the CDC’s 2025 plan to establish a zoonosis response team (CDC, 2025). Regular updates ensure the public remains informed with the latest scientific findings, while a crisis communication plan ensures swift, coordinated messaging during health emergencies. By proactively engaging with media and stakeholders, the council helps maintain trust and prevent panic.

## Structure of the Zoonosis Council

**Figure 1.**
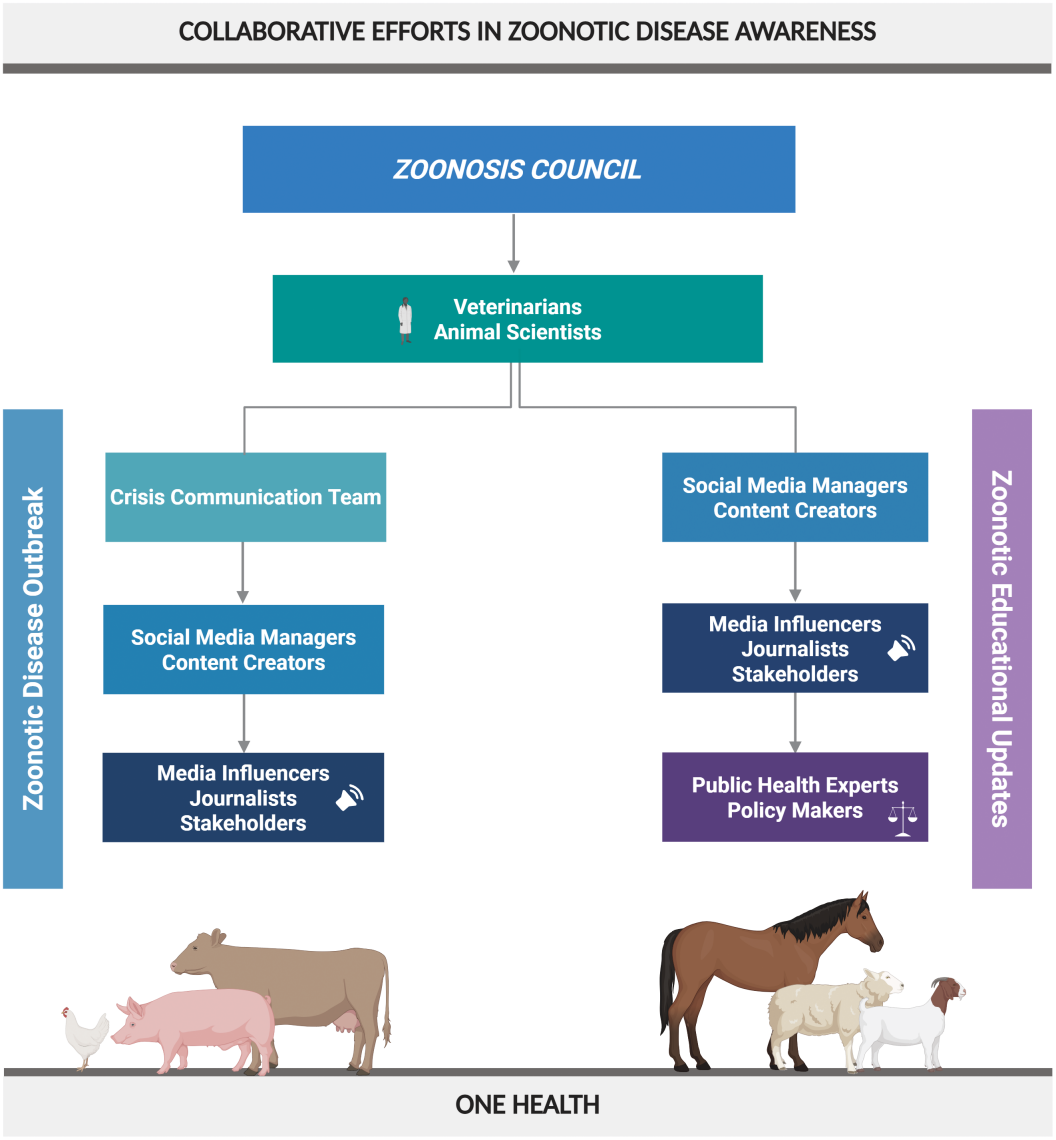
A unified zoonotic disease council combats misinformation with strategic, evidence-based communication. Veterinarians and animal scientists guide a crisis communication team, working with public health, government, and media to ensure clear messaging. Social media managers create engaging content for influencers, journalists, and stakeholders to amplify. Regular updates from experts promote accurate zoonotic disease education and prevention, aligning with One Health goals and regulatory needs.

The Zoonosis Council will be composed of interdisciplinary teams, coordinated through a central leadership structure and project management unit (Figure 1). Council members will include veterinarians, epidemiologists, public health experts, animal scientists, Extension specialists, ecologists, economists, and science communicators. Each critical area of zoonosis communication will be supported by a dedicated project manager, ensuring expert oversight and streamlined efforts across domains.

### Expert Locator Team

The Expert Locator Team will be responsible for identifying and engaging subject matter experts related to the current pressing zoonotic disease outbreak. This team will actively source experts who can provide the foundational scientific knowledge on specific zoonotic diseases and ensure the Council’s efforts remain evidence-based. The Expert Locator Team will maintain ongoing relationships with a network of specialists, regularly consulting them to verify content accuracy and relevance. This team will play a continuous role throughout the lifecycle of a zoonotic event, updating the expert roster and bringing in new voices as emerging issues arise.

### Disease Identification and Management Team

The Disease Identification and Management Team will work in collaboration with experts in animal health, veterinary medicine, and public health to develop educational content on zoonotic disease identification and management. The team will create materials on prevention strategies, diagnostic protocols, livestock management, biosecurity, and treatment options. Additionally, this team will partner with the Social Media Campaign Team to convert technical information into engaging, user-friendly content for broad public dissemination.

### Key Stakeholder Outreach and Influencer Engagement Team

The Key Stakeholder Outreach and Influencer Engagement Team, led by an outreach manager, will build relationships with industry leaders, social media influencers, and media outlets. This team will coordinate strategic outreach efforts, including media appearances, podcast interviews, and conference engagements, to amplify the Council’s messaging across agriculture, health, and policy sectors.

### Social Media Campaign Team

**Figure 2.**
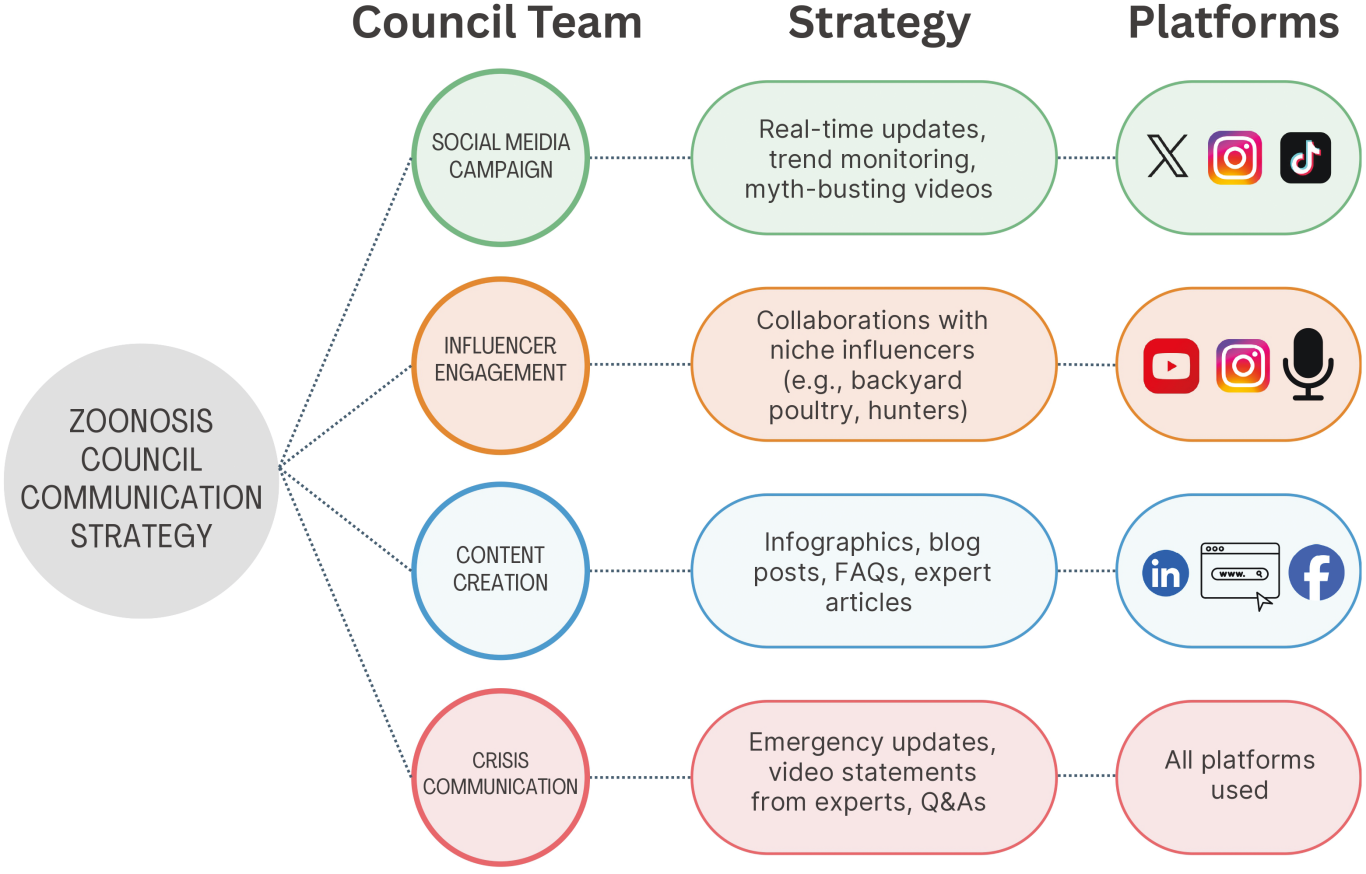
The Zoonosis Council uses a multi-faceted communication strategy, with dedicated teams delivering targeted messages across platforms. This includes real-time updates and myth-busting videos via social media, partnerships with niche influencers, educational content like infographics and expert articles, and crisis communication. Each message is tailored to the most effective platforms, from TikTok and Instagram to LinkedIn and Facebook, to ensure timely and accurate outreach.

The Social Media Campaign Team will manage digital communication efforts across platforms such as X, Facebook, Instagram, and TikTok, ensuring the distribution of fact-based, timely updates about zoonotic diseases (Figure 2). This team will monitor trending topics, track engagement metrics, and adjust messaging strategies to enhance public reach, credibility, and trust in real time.

### Crisis Communication Team

The Crisis Communication Team will serve as the rapid-response arm of the Council during zoonotic disease outbreaks. This media-trained team will be responsible for delivering fast, accurate updates to the public, ensuring alignment with the work of other Council teams. The Crisis Communication Team will prioritize consistency in messaging paired with timely content delivery across traditional and digital communication platforms.

## Implementation and Funding

Initial funding for the Zoonosis Council could be sought through USDA-NIFA One Health programs, CDC’s Crisis Response Cooperative Agreements, or carefully structured public–private partnerships with agricultural and veterinary stakeholders. Coordination will be managed by a centralized project lead and supported by cross-functional working groups, regular communication sprints, and annual evaluations to assess progress, outreach impact, and public trust. While initial support may involve diverse funding streams, the Council’s long-term objective is to operate as an independently governed entity, with strict policies in place to prevent donor influence on educational content and strategic priorities.

## EXAMPLE: The Zoonosis Council in Action

Public perception of the 2025 HPAI outbreak appears to have been shaped by a combination of media coverage, inconsistent messaging, and circulating misinformation, contributing to public uncertainty and economic strain within the poultry industry. A structured, science-based communication approach can improve public understanding and response during zoonotic outbreaks by promoting consistency, transparency, and trust. The proposed Zoonosis Council is designed to serve this purpose by coordinating interdisciplinary expertise and delivering timely, audience-specific messaging before, during, and after zoonotic disease events.

## Impacts of the Zoonosis Council During Critical Outbreak Periods

### Real-time, fact-based communication

The Zoonosis Council will provide a single, verified source of real-time updates to news outlets, social media, and public briefings, reducing confusion caused by delayed or inconsistent information.

### Targeted misinformation response

One of the greatest challenges of the HPAI outbreak has been the spread of misinformation across social media and news outlets. The Council’s social media campaign team will actively monitor trending narratives and respond quickly with engaging, scientifically accurate content, including fact-check posts, infographics, and expert-led videos, to correct public misconceptions before they gain traction.

### Audience-specific outreach

Traditional communication often centers on industry professionals and government agencies, leaving out smaller yet highly engaged communities. The Council will use inclusive strategies and directly engage key influencers and stakeholders in niche groups. Specific outreach strategies during an HPAI outbreak will target:

#### Backyard bird enthusiasts and hobby farmers.

Partnering with homesteading and small producer influencers on TikTok, YouTube, and Instagram to share best biosecurity practices, dispel myths, and encourage responsible flock management.

#### Hunting and outdoor communities.

Partnering with hunting influencers and conservation groups to educate about the risks of migratory bird transmission and how hunters can help prevent the spread of HPAI through proper handling and reporting.

### Bridging the gap between scientists and the public

Many official reports on HPAI have been overly technical or buried in agency websites, making them inaccessible to the general public. The Council’s content creation team will simplify complex veterinary and epidemiological data into digestible, engaging formats (short-form videos, visual explainers, and Q&A sessions with experts) to best address the public sphere.

### Crisis communication without fearmongering

Government agencies and media outlets have contributed to public uncertainty by alternating between alarmist warnings and vague reassurances, undermining public trust. The Council’s crisis communication team will aim to strike a balance, acknowledging real risks without inducing panic. Communications will focus on:

- The scientific likelihood of zoonotic transmission.
- Effective biosecurity measures to protect poultry flocks.
- How consumers can confidently purchase poultry products without fear.

### Public education beyond the outbreak

Rather than operating solely in response to crises, the Council will maintain ongoing public education campaigns on zoonotic diseases. Promoting One Health principles and proactive disease prevention strategies can build public trust and reduce panic-driven responses when future outbreaks occur.

## The Bottom Line

Misinformation during zoonotic outbreaks undermines public health and agricultural stability. The proposed Zoonosis Council offers a forward-looking, interdisciplinary strategy to improve communication, engage diverse audiences, and promote science-based decision-making. By learning from global models and leveraging Cooperative Extension and digital media, this Council can help ensure future zoonotic outbreaks are met with clarity, not confusion.

## About the Authors

**Luke M. Sanders** is an undergraduate student in Animal Science at Oklahoma State University, where he conducts research under the mentorship of Dr. Riley Messman. His research centers on the interactions between fungi and Bovine Respiratory Disease in feedlot cattle, aiming to improve livestock health management. Outside of research, Luke is deeply involved in youth development programs such as 4-H and FFA, with a particular focus on Veterinary Science contests and related projects. At Oklahoma State, he is an active participant in the Ferguson College of Agriculture and the Department of Animal and Food Sciences, contributing to various initiatives within these communities.

**Mamie-Cate Haydon** is a first-year master’s student at Oklahoma State University, specializing in Bovine Reproductive Physiology under the mentorship of Dr. Riley Messman. Raised on a sixth-generation cow-calf operation in Weatherford, Texas, she developed a strong appreciation for land and livestock stewardship. Her experience as a ruminant embryologist deepened her appreciation and fueled her desire to understand the various significant factors driving success in livestock production and reproduction. Her research focuses on assessing the needs and current management practices of beef cow-calf producers, particularly analyzing the barriers to adopting assisted reproductive technologies (ART). Mamie-Cate’s ultimate goal is to aid in bridging the gap between innovation and practical producer application to enhance sustainable beef cattle production and reproductive efficiency.

**Dr. Riley Messman** is an assistant professor in the Animal and Food Sciences department at Oklahoma State University, where she holds responsibilities in teaching and research. Her research focuses on how the maternal uterine environment affects offspring’s health and performance. She’s currently studying the microbiome of the maternal reproductive tract, implications of neonatal microbial inoculation, and upper respiratory tract microbiome fluctuations during early life. At the undergraduate level, Dr. Messman teaches Introduction to Animal Science and Livestock Health & Disease courses. She aims to educate future veterinarians, producers, and industry leaders to identify health issues and use innovative management practices to improve overall herd health.

## Author Note

If you are interested in joining the effort to develop a Zoonosis council, please contact the corresponding author to get involved.
